# Book Reviews

**Published:** 1900-09

**Authors:** 


### *BOOK REVIEWS, PAMPHLETS, EXCHANGES.*

### *BOOK REVIEWS.*

[Contributions solicited for review.]

GALL-STONES AND DISEASES OF THE GALL-BLADDER AND NERVOUS SYMPTOMS RESULTING THEREFROM. Based on an Experience of Forty-six Cases. By Dr. Edwin Rickett, Cincinnati, Ohio. American Medical Association, Atlantic City meeting.

1. Gall-stones are found in the excretory apparatus of the liver.

2. Clinically speaking, there is recognized by operators marked nervous manifestations of a most detrimental kind.

3. Just how this is caused has not so far been satisfactorily explained.

4. Gall-stones are products that may originate in the intrahepatic ducts, the hepatic duct, the cystic duct, the gall-bladder and the common bile duct.

5. The contents of the gall-bladder, with or without gall-stones, may become infected by the bacterium coli commune or a pyogenic streptococcus by entering the biliary passages from the duodenum or through the blood of the portal vein.

6. Gall-stones should be removed as soon as diagnosed.

7. Distended gall-bladders that fail to empty themselves by well selected internal medication, with massage through the abdominal wall, should be drained by means of cholecystotomy.

---

STIMSON'S OPERATIVE SURGERY. A Manual of Operative Surgery. By Lewis A. Stimson, B.A., M.D., Professor of Surgery in Cornell University Medical College. New (4th) and thoroughly revised edition. In one royal 12mo volume of 581 pages, with 293 illustrations. Just ready. Cloth, $3.00, net. Lea Brothers & Co., Philadelphia and New York.

The reputation of this work has been sustained through four editions. The last edition is a thorough revision, and the subject of operative surgery is concisely set forth and illustrated where illustrations are necessary. The book consists of 581 pages and has been revised up to the date of publication. It is commended to those who are in need of a reliable, inexpensive work on this subject.

---

KING'S MANUAL OF OBSTETRICS. New (8th) Edition. A Manual of Obstetrics. By A. F. A. King, M.D., Professor of Obstetrics and Diseases of Women in the Medical Department of the Columbian University, Washington, D. C., and in the University of Vermont, etc. In one 12mo volume of 612 pages, with 264 illustrations. Cloth, $2.50, net. Lea Brothers & Co., Publishers, Philadelphia and New York.

King's Obstetrics has enjoyed popularity as a text-book for many years. The present edition—the eighth—is an enlargement of the older ones, and is carefully brought up to date to meet the advances in this field of medicine. It is well illustrated and printed, and sells for the moderate sum of $2.50. To those who are not already familiar with the book it is heartily recommended to their consideration.

---

ATLAS AND EPITOME OF DISEASES CAUSED BY ACCIDENTS. By Dr. Ed. Golebiewski of Berlin. Translated from the German by Pearce Bailey, M.D., New York. 40 colored plates and 143 illustrations in black. Philadelphia. W. B. Saunders & Co. 1900.

This work serves to illustrate the various sequels of traumatism and is of interest not only from a professional standpoint but also in its relation to accident insurance. There are very few works of this character and their work can be readily appreciated. The book is splendidly illustrated in colors, in black and white skiagraphs. It sells for $3.50.

